# 2-[(2,4,6-Tri­methyl­benzene)­sulfon­yl]phthalazin-1(2*H*)-one: crystal structure, Hirshfeld surface analysis and computational study

**DOI:** 10.1107/S2056989020005101

**Published:** 2020-04-21

**Authors:** David Chukwuma Izuogu, Jonnie Niyi Asegbeloyin, Mukesh M. Jotani, Edward R. T. Tiekink

**Affiliations:** a Department of Pure and Industrial Chemistry, University of Nigeria, Nsukka 410001, Enugu State, Nigeria; b Department of Chemistry, Graduate School of Science, Tohoku University, 6-3 Aza-aoba, Aramaki, Sendai 980-8578, Japan; c Department of Chemistry, University of Cambridge, Lensfield Road, CB2 1EW, UK; d Department of Physics, Bhavan’s Sheth R. A. College of Science, Ahmedabad, Gujarat 380001, India; e Research Centre for Crystalline Materials, School of Science and Technology, Sunway University, 47500 Bandar Sunway, Selangor Darul Ehsan, Malaysia

**Keywords:** crystal structure, phthalazinone, Hirshfeld surface analysis

## Abstract

The X-ray crystal structure of the title phthalazin-1-one derivative, C_17_H_16_N_2_O_3_S {systematic name: 2-[(2,4,6-tri­methyl­benzene)­sulfon­yl]-1,2-di­hydro­phthalazin-1-one}, features a tetra­hedral sulfoxide-S atom, connected to phthalazin-1-one and mesityl residues. The dihedral angle [83.26 (4)°] between the organic substituents is consistent with the mol­ecule having the shape of the letter *V*. In the crystal, phthalazinone-C_6_-C—H⋯O(sulfoxide) and π(phthalazinone-N_2_C_4_)–π(phthalazinone-C_6_) stacking [inter-centroid distance = 3.5474 (9) Å] contacts lead to a linear supra­molecular tape along the *a*-axis direction; tapes assemble without directional inter­actions between them. The analysis of the calculated Hirshfeld surfaces confirm the importance of the C—H⋯O and π-stacking inter­actions but, also H⋯H and C—H⋯C contacts. The calculation of the inter­action energies indicate the importance of dispersion terms with the greatest energies calculated for the C—H⋯O and π-stacking inter­actions.

## Abstract 2

The mol­ecule in the title crystal has the shape of the letter *V* with the dihedral angle between the phthalazin-1-one and mesityl residues being 83.26 (4)°. Mol­ecules assemble into a linear, supra­molecular tape by phthalazinone-C_6_-C—H⋯O(sulfoxide) and π(phthalazinone-N_2_C_4_)–π(phthalazinone-C_6_) stacking inter­actions.

## Chemical context   

Phthalazin-1(2*H*)-one derivatives are a group of di­aza­heterobicycles that are noteworthy for their inter­esting medicinal applications. Thus, this class of compound has been reported to possess a wide variety of biological properties such as anti-diabetic (Mylari *et al.*, 1992 ▸), anti-cancer (Menear *et al.*, 2008 ▸ *)*, anti-inflammatory and analgesic (Pakulska *et al.*, 2009 ▸), anti-histamine (Procopiou *et al.*, 2011 ▸), anti-hypertensive and anti-thrombotic (Cherkez *et al.*, 1986 ▸) activities. Some N-substituted phthalazinones have attracted attention as a result of their potential role as anti-asthmatic agents (Ukita *et al.*, 1999 ▸), their ability to inhibit thromboxane A2 (TXA2) synthetase and to induce bronchodialation (Yamaguchi *et al.*, 1993 ▸). At the present time, a number of phthalazin-1(2*H*)-one-based drugs are in use (Wu *et al.*, 2012 ▸; Teran *et al.*, 2019 ▸). A number of reaction pathways to the phthalazinone skeleton are known, notable among which include multi-step reactions involving cyclo­condensation reactions of phthalic anhydrides, phthalimides, phthalaldehydic acid or 2-acyl­benzoic acids with substituted hydrazines, in the presence of appropriate catal­ysts (Haider & Holzer, 2004 ▸). The conversion of phthalimides *via* Friedel–Crafts conditions or with organometallics to 2-keto benzoic acid hydrazides or 3,3-disubstituted indolin­ones, which are viable inter­mediates to substituted phthalazin-1(2*H*)-ones, have also been reported (Ismail *et al.*, 1984 ▸; Chun *et al.*, 2004 ▸) . Several other synthetic routes, involving various inter­mediates, have also been reported (Mylari *et al.*, 1991 ▸; Yamaguchi *et al.*, 1993 ▸; Acosta *et al.*, 1995 ▸; Bele & Darabantu, 2003 ▸; Mahmoodi & Salehpour, 2003 ▸; Cockcroft *et al.*, 2006 ▸; Del Olmo *et al.*, 2006 ▸). In an earlier communication (Asegbeloyin *et al.*, 2018 ▸), the dysprosium(III)-catalysed conversion of 2-{[2-(phenyl­sulfon­yl)hydrazinyl­idene] meth­yl}benzoic acid to 2-(phenyl­sulfon­yl)phthalazin-1-(2*H*)-one was described. In the present study, the title compound, 2-[(2,4,6-tri­methyl­benzene)­sulfon­yl]-1,2-di­hydro­phthalazin-1-one, (I), was obtained by the catalytic conversion of 2-{[2-(2,4,6-tri­methyl­phenyl­sulfon­yl)hydrazinyl­idene]meth­yl}benzoic acid. Herein, the crystal and mol­ecular structures of (I) are described as is a detailed analysis of the mol­ecular packing by an evaluation of the calculated Hirshfeld surfaces augmented by a computational chemistry study.
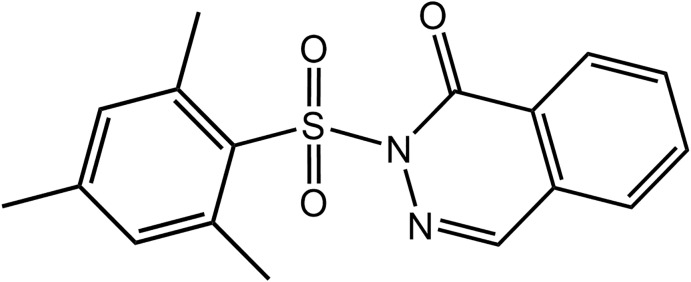


## Structural commentary   

**Figure 1:**
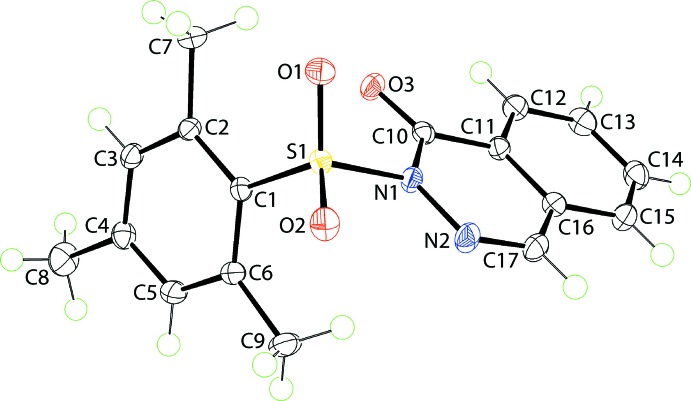
The mol­ecular structures of (I) showing the atom-labelling scheme and displacement ellipsoids at the 70% probability level.

The mol­ecule of (I), Fig. 1 ▸, may be conveniently described as a central SO_2_ residue with mesityl and phthalazin-1-one substituents. The geometry about the S1 atom is distorted tetra­hedral with the range of angles subtended at S1 being a narrow 103.58 (6)° for N1—S1—C1, involving the singly-bonded N1 and C1 atoms, to a wide 118.39 (6)°, for O1—S1—O2, involving the doubly-bonded sulfoxide-O1, O2 atoms. The organic residues lie to the opposite side of the mol­ecule to the SO_2_ residue, forming dihedral angles of 67.35 (4)° [phthalazin-1-one with r.m.s. deviation = 0.0105 Å] and 49.79 (6)° [mesit­yl]. The dihedral angle between the organic residues of 83.26 (4)° indicates a close to orthogonal relationship. The N2—N1—C10—O3 torsion angle of −179.88 (12)° indicates a co-planar arrangement for these atoms, which allows for the close approach of the N2 and O3 atoms, *i.e*. 2.6631 (15) Å, suggestive of a stabilizing contact (Nakanishi *et al.*, 2007 ▸). Globally, the mol­ecule has the shape of the letter *V*. Within the hetero-ring of the phthalazin-1-one substituent, the N1—N2 bond length is 1.3808 (15) Å and C10—N1 = 1.4003 (17) Å. In each of the C17=N2 [1.2911 (18) Å] and C10=O3 [1.2175 (15) Å] bonds, double-bond character is noted. The bond angles about the N1 atom are non-symmetric, with the endocyclic N2—N1—C10 angle of 126.97 (11) Å being significantly wider than the exocyclic N2—N1—S1 [113.93 (9) Å] and C10—N1—S1 [118.89 (8) Å] angles.

## Supra­molecular features   

**Figure 2:**
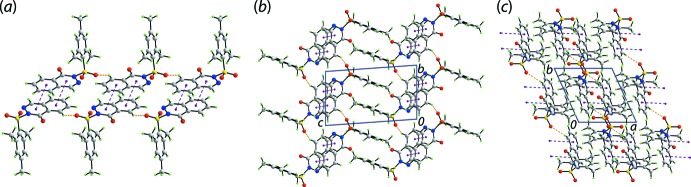
Mol­ecular packing in the crystal of (I): (*a*) supra­molecular tape sustained by phthalazinone-C—H⋯O(sulfoxide) and π(phthalazinone)–π(phthalazinone) stacking interactions shown as orange and purple dashed lines, respectively, (*b*) a view of the unit-cell contents down the *a* axis showing the inter-digitation of tapes and (*c*) a view of the unit-cell contents down the *c* axis showing the stacking of assemblies of (*b*) along the *a*-axis direction.

The formation of a supra­molecular tape sustained by phthal­a­zinone-C_6_-C—H⋯O(sulfoxide) contacts, Table 1 ▸, and π(phthalazinone)–π(phthalazinone) stacking is the main feature of the mol­ecular packing in the crystal of (I), Fig. 2 ▸(*a*). The π-stacking occurs between centrosymmetrically related phthalazinone rings, *i.e*. between the N_2_C_4_ and C_6_^i^ rings with an inter-centroid distance = 3.5474 (9) Å, angle of inclination = 1.17 (7)° for symmetry operation (i) 1 − *x*, 1 − *y*, 2 − *z*. As shown in Fig. 2 ▸(*b*), the tapes inter-digitate along the *c*-axis direction allowing for putative π-stacking between mesityl rings but, the inter-centroid separation is long at 4.1963 (8) Å. The assemblies shown in Fig. 2 ▸(*b*) stack along the *a*-axis direction, again without directional inter­actions between them, Fig. 2 ▸(*c*).

## Hirshfeld surface analysis   

**Figure 3:**
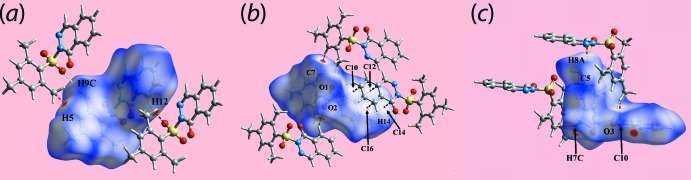
(*a*)–(*c*) Three views of Hirshfeld surface mapped over *d*_norm_ for (I) in the range −0.128 to + 1.298 arbitrary units. The inter­molecular C-H⋯O and short inter­atomic C⋯C contacts are represented with black dashed lines, and the short inter­atomic H⋯H, O⋯H/H⋯O and C⋯H/H⋯C contacts with sky-blue, yellow and red dashed lines, respectively.

**Figure 4:**
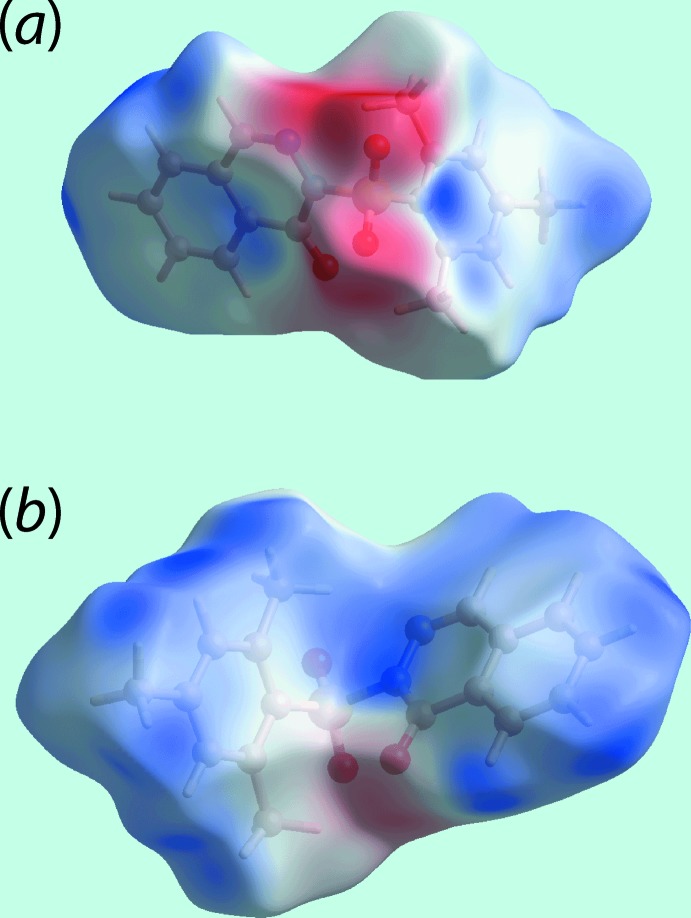
(*a*) and (*b*) Two views of the calculated electrostatic potential mapped onto the Hirshfeld surface within the isosurface range −0.093 to 0.040 atomic units. The red and blue regions represent negative and positive electrostatic potentials, respectively.

In order to probe the inter­actions between mol­ecules of (I) in the crystal, the Hirshfeld surfaces and two-dimensional fingerprint plots were calculated with the program *Crystal Explorer 17* (Turner *et al.*, 2017 ▸) using established procedures described by Tan *et al.* (2019 ▸). In addition to the bright-red spots appearing near the sulfoxide-O2 and phthalazinone-H12 atoms on the Hirshfeld surface in Fig. 3 ▸(*a*),(*b*), the presence of diminutive red spots near methyl-C7 and benzene-H5 are indicative of inter­molecular C—H⋯C contacts as C—H⋯π contacts are not preferred because of the *V*-shaped mol­ecular geometry of (I). Also, the group of faint-red spots near alternate carbon atoms C10, C12, C14 and C16 of the phthalazinone-C_6_ ring on the *d*_norm_-mapped Hirshfeld surface in Fig. 3 ▸(*b*) is indicative of short intra-chain C⋯C contacts [Table 2 ▸ and Fig. 2 ▸(*a*)] and is consistent with the significant contribution from π–π stacking between centrosymmetrically related phthalazinone-N_2_C_4_ and C_6_ rings, encompassing connections between phthalazinone-C_6_ rings [3.6657 (9) Å with angle of inclination = 0.03 (7)°]. The involvement of the methyl-C8 atom in C—H⋯O [to provide links between the chains shown in Fig. 2 ▸(*b*)] and C—H⋯C contacts, Table 2 ▸, is highlighted in Fig. 3 ▸(*c*). The blue and red regions corres­ponding to positive and negative electrostatic potentials, respectively, on the Hirshfeld surface mapped over electrostatic potential shown in Fig. 4 ▸ represent the involvement of different atoms in the inter­molecular inter­actions in the crystal.

**Figure 5:**
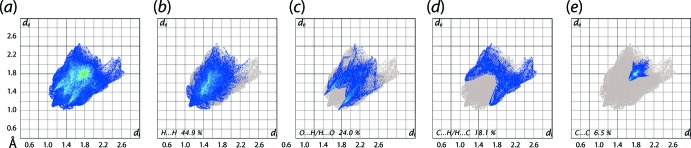
(*a*) The overall two-dimensional fingerprint plots for (I), and those delineated into (*b*) H⋯H, (*c*) O⋯H/H⋯O, (*d*) C⋯H/H⋯C and (*e*) C⋯C contacts.

The overall two-dimensional fingerprint plots for (I) and those delineated into H⋯H, O⋯H/H⋯O, C⋯H/H⋯C and C⋯C contacts are illustrated in Fig. 5 ▸(*a*)–(*e*), respectively; the percentage contributions from the different inter­atomic contacts to the Hirshfeld surfaces are summarized in Table 3 ▸. A short inter­atomic H⋯H contact involving the phthalazinone-H12 and methyl-H9*A* atoms, Table 2 ▸, appears as a small peak at *d*_e_ + *d*_i_ ∼2.2 Å in the fingerprint plot delineated into H⋯H contacts, Fig. 5 ▸(*b*). In the fingerprint plot delineated into O⋯H/H⋯O contacts illustrated in Fig. 5 ▸(*c*), a pair of forceps-like tips at *d*_e_ + *d*_i_ ∼2.3 Å, indicate the inter­molecular C—H⋯O inter­action involving the phthalazinone-H12 and sulfoxide-O2 atoms, whereas the other inter­atomic O⋯H/H⋯O contacts are merged within the plot and appear as a pair of intense blue spikes at *d*_e_ + *d*_i_ ∼2.8 Å. Despite the observation that inter­molecular C—H⋯π contacts are usually preferred by methyl groups, none are found involving those substituted at (C1–C6) benzene ring in the crystal due to the *V*-shaped geometry. Rather, the involvement of methyl-C7 and H5*A* atoms, and benzene-C5 and H7*C* atoms [to provide links between the chains shown in Fig. 2 ▸(*b*)] in C—H⋯C inter­actions, Table 2 ▸, are characterized as the pair of forceps-like flat tips about *d*_e_ + *d*_i_ ∼2.8 Å in the fingerprint plot delineated into C⋯H/H⋯C contacts, Fig. 5 ▸(*d*). The presence of π–π stacking inter­actions between symmetry-related phthalazinone-N_2_C_4_ and C_6_ rings is also evident as the arrow-shaped distribution of points around *d*_e_, *d*_i_ ∼1.8 Å in the fingerprint plot delineated into C⋯C contacts, Fig. 5 ▸(*e*). The contribution from other inter­atomic contacts, summarized in Table 2 ▸, show a negligible effect on the calculated Hirshfeld surface of (I).

## Computational chemistry   

The pairwise inter­action energies between the mol­ecules within the crystal of (I) were calculated by summing up four energy components, comprising electrostatic (*E*_ele_), polarization (*E*_pol_), dispersion (*E*_dis_) and exchange–repulsion (*E*_rep_) following Turner *et al.* (2017 ▸). The energies were obtained by using the wave function calculated at the B3LYP/6-31G(*d*,*p*) level of theory. The nature and strength of the inter­molecular inter­actions in terms of their energies are qu­anti­tatively summarized in Table 4 ▸, where it is clear that the dispersive component makes the major contribution to the inter­action energies in the crystal in the absence of conventional hydrogen bonding. It is revealed from the inter­action energies listed in Table 4 ▸, that the π–π stacking inter­action between phthalazinone-N_2_C_4_ and C_6_ rings and the short inter­atomic O1⋯H14 contact have the greatest energy. The short inter­atomic C5⋯H7*C*, O3⋯H8*A* and C10⋯H8*A* contacts also have significant inter­action energies due to their participation in inversion-related contacts. Lower energies, compared to above inter­actions, are calculated for the H12⋯H9*A*, C7⋯H5 and O1⋯H9*C* contacts.

**Figure 6:**
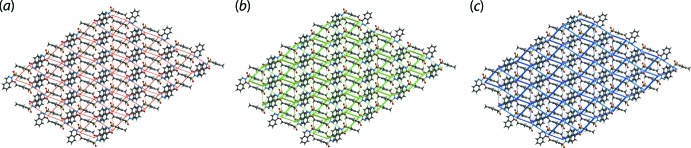
Perspective views of the energy frameworks calculated for (I), showing the (*a*) electrostatic force, (*b*) dispersion force and (*c*) total energy. The radii of the cylinders are proportional to the relative strength of the corresponding energies and were adjusted to the same scale factor of 50 with a cut-off value of 3 kJ mol^−1^ within 4 × 4 × 4 unit cells.

Fig. 6 ▸ illustrates the magnitudes of inter­molecular energies represented graphically by energy frameworks to highlight the supra­molecular architecture of the crystal through cylinders joining the centroids of mol­ecular pairs using red, green and blue colour codes for the components *E*_ele_, *E*_disp_ and *E*_tot_, respectively. The images emphasize the importance of dispersion inter­actions in the mol­ecular packing.

## Database survey   

**Figure 7:**
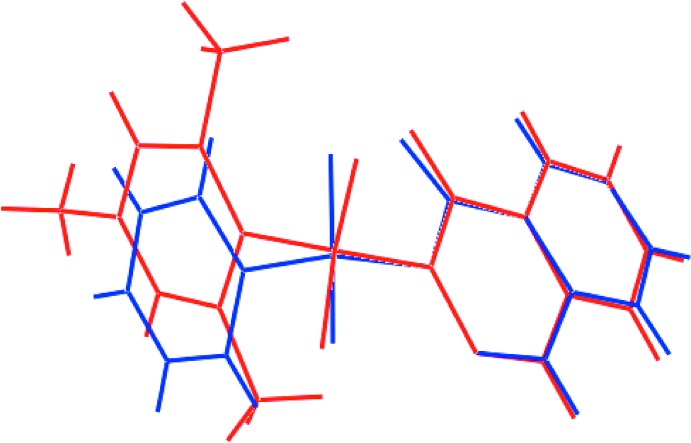
An overlay diagram for (I) (red image) and (II) (blue). The mol­ecules have been overlapped so the hetero-rings are coincident.

There is only a single direct analogue to (I) in the crystallographic literature, namely 2-(phenyl­sulfon­yl)phthalazin-1(2*H*)-one (Asegbeloyin *et al.*, 2018 ▸), (II). A comparison of key geometric parameters for (I) and (II) is given in Table 5 ▸. The data in Table 5 ▸ confirm the closeness of the salient bond lengths, but also show significant differences in the torsion angles about the N1—S1 and C1—S1 bonds, *i.e*. by up to 18 and 8°, respectively. These conformational differences are highlighted in the overlay diagram of Fig. 7 ▸ and in the dihedral angles between the aromatic residues of 83.26 (4) and 78.12 (4)° for (I) and (II), respectively.

## Synthesis and crystallization   

2-{[2-(2,4,6-Tri­methyl­phenyl­sulfon­yl)hydrazinyl­idene]meth­yl}benzoic acid (III) was obtained by a method reported earlier (Asegbeloyin *et al.*, 2018 ▸). Compound (I) was obtained from the following reaction. An ethanol solution (10 ml) of Dy(O_2_CCH_3_)_3_·4H_2_O (Wako Chemicals, Japan; 1 mmol, 411.692 mg) was added with constant stirring to an ethanol solution (20 ml) of (III) (1,039.2 mg, 3 mmol). The resulting mixture was refluxed for 3 h in an oil bath. The obtained colourless solution was concentrated to afford a colourless precipitate, which was filtered, dried under suction and further dried *in vacuo* over CaCl_2_. The precipitates were dissolved in ethanol, the resultant colourless solution was filtered and left at room temperature for 48 h to obtain colourless crystals of (I).

## Refinement   

Crystal data, data collection and structure refinement details are summarized in Table 6 ▸. The carbon-bound H atoms were placed in calculated positions (C—H = 0.95–0.98 Å) and were included in the refinement in the riding-model approximation, with *U*_iso_(H) set to 1.2–1.5*U*_eq_(C).

## Supplementary Material

Crystal structure: contains datablock(s) I, global. DOI: 10.1107/S2056989020005101/lh5956sup1.cif

Structure factors: contains datablock(s) I. DOI: 10.1107/S2056989020005101/lh5956Isup2.hkl

Supporting information file. DOI: 10.1107/S2056989020005101/lh5956Isup3.cml

CCDC reference: 1996401

Additional supporting information:  crystallographic information; 3D view; checkCIF report

## Figures and Tables

**Table 1:** Hydrogen-bond geometry (Å, °)

*D*—H⋯*A*	*D*—H	H⋯*A*	*D*⋯*A*	*D*—H⋯*A*
C12—H12⋯O2^i^	0.95	2.49	3.3395 (18)	149

Symmetry code: (i) 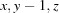.

**Table 2:** A summary of short inter­atomic contacts (Å) in (I)*^*a*^*

Contact	Distance	Symmetry operation
C10⋯C14	3.345 (2)	1 − *x*, 1 − *y*, 2 − *z*
C12⋯C16	3.351 (2)	1 − *x*, 1 − *y*, 2 − *z*
O1⋯H9*C*	2.58	1 + *x*, *y*, *z*
O1⋯H14	2.61	1 − *x*, 1 − *y*, 2 − *z*
O3⋯H8*A*	2.60	−*x*, 1 − *y*, 1 − *z*
C5⋯H7*C*	2.78	1 − *x*, 2 − *y*, 1 − *z*
C7⋯H5	2.61	1 + *x*, *y*, *z*
C10⋯H8*A*	2.79	−*x*, 1 − *y*, 1 − *z*
H12⋯H9*A*	2.20	*x*, −1 + *y*, *z*

Notes: (*a*) The inter­atomic distances are calculated in *Crystal Explorer 17* (Turner *et al.*, 2017 ▸) whereby the *X*—H bond lengths are adjusted to their neutron values; (*b*) these inter­actions correspond to conventional hydrogen bonds.

**Table 3:** Percentage contributions to inter­molecular contacts on the Hirshfeld surface calculated for (I)

Contact	Percentage contribution
H⋯H	44.9
O⋯H/H⋯O	24.0
C⋯H/H⋯C	18.1
C⋯C	6.5
N⋯H/H⋯ N	4.0
C⋯O/O⋯C	1.1
C⋯N/N⋯C	0.7
N⋯N	0.4
C⋯S/S⋯C	0.2

**Table 4:** A summary of inter­action energies (kJ mol^−1^) calculated for (I)

Contact	R (Å)	*E*_ele_	*E*_pol_	*E*_dis_	*E*_rep_	*E*_tot_
*Cg*(N_2_C_4_)⋯*Cg*(C_6_)^i^ +						
*Cg*(C_6_)⋯*Cg*(C_6_)^i^ +	8.12	−28.9	−5.0	−64.7	48.2	−60.8
O1⋯H14^i^						
C5⋯H7*C* ^ii^	7.84	−21.3	−5.5	−60.9	43.5	−52.8
O3 ⋯H8*A* ^iii^ +						
C10 ⋯H8*A* ^iii^	7.54	−10.7	−2.0	−56.8	32.6	−42.1
C12—H12⋯O2^iv^ +						
H12⋯H9*A* ^iv^	8.17	−4.4	−4.6	−20.5	18.0	−14.8
O1⋯H9*C* ^v^ +						
C7⋯H5^v^	7.98	−3.1	−2.0	−16.9	14.2	−10.6

Symmetry codes: (i) 1 − *x*, 1 − *y*, 2 − *z*; (ii) 1 − *x*, 2 − *y*, 1 − *z*; (iii) − *x*, 1 − *y*, 1 − *z*; (iv) *x*, −1 + *y*, *z*; (v) 1 + *x*, *y*, *z*.

**Table 5:** A comparison of key geometric parameters (Å, °) for (I) and (II)

	(I)	(II)
N1—N2	1.3808 (15)	1.384 (2)
C10—O3	1.2175 (15	1.212 (3)
C10—N1	1.4003 (17)	1.406 (2)
C17—N2	1.2911 (18)	1.283 (2)
N2⋯O2	2.6631 (15)	2.6394 (19)
N2—N1—S1—O1	120.33 (10)	138.71 (12)
N2—N1—S1—O2	−5.52 (11)	9.59 (13)
N1—S1—C1—C2	−111.55 (11)	−103.95 (16)
N1—S1—C1—C6	69.70 (11)	76.49 (17)

**Table 6:** Experimental details

Crystal data	
Chemical formula	C_17_H_16_N_2_O_3_S
*M*_r_	328.38
Crystal system, space group	Triclinic, *P* 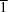
Temperature (K)	100
*a*, *b*, *c* (Å)	7.9782 (4), 8.1711 (5), 12.6661 (7)
α, β, γ (°)	92.214 (2), 93.423 (1), 114.274 (1)
*V* (Å^3^)	749.55 (7)
*Z*	2
Radiation type	Mo *K*α
μ (mm^−1^)	0.23
Crystal size (mm)	0.38 × 0.12 × 0.08

Data collection	
Diffractometer	Bruker APEXII CCD
Absorption correction	Multi-scan (*SADABS*; Sheldrick, 1996 ▸)
*T*_min_, *T*_max_	0.924, 1.000
No. of measured, independent and observed [*I* > 2σ(*I*)] reflections	11665, 5913, 4625
*R*_int_	0.027
(sin θ/λ)_max_ (Å^−1^)	0.804

Refinement	
*R*[*F* ^2^ > 2σ(*F* ^2^)], *wR*(*F* ^2^), *S*	0.048, 0.122, 1.02
No. of reflections	5913
No. of parameters	211
H-atom treatment	H-atom parameters constrained
Δρ_max_, Δρ_min_ (e Å^−3^)	0.59, −0.39

Computer programs: *APEX2* and *SAINT* (Bruker, 2002 ▸), *SHELXT* (Sheldrick, 2015*a* ▸), *SHELXL2018/3* (Sheldrick, 2015*b* ▸), *ORTEP-3 for Windows* (Farrugia, 2012 ▸), *DIAMOND* (Brandenburg, 2006 ▸) and *publCIF* (Westrip, 2010 ▸).

## supplementary crystallographic information

### Crystal data

**Table:**

C_17_H_16_N_2_O_3_S	*Z* = 2
*M**_r_* = 328.38	*F*(000) = 344
Triclinic, *P*1	*D*_x_ = 1.455 Mg m^−^^3^
*a* = 7.9782 (4) Å	Mo *K*α radiation, λ = 0.71073 Å
*b* = 8.1711 (5) Å	Cell parameters from 5241 reflections
*c* = 12.6661 (7) Å	θ = 2.7–34.7°
α = 92.214 (2)°	µ = 0.23 mm^−^^1^
β = 93.423 (1)°	*T* = 100 K
γ = 114.274 (1)°	Prism, colourless
*V* = 749.55 (7) Å^3^	0.38 × 0.12 × 0.08 mm

### Data collection

**Table:**

Bruker APEXII CCD diffractometer	4625 reflections with *I* > 2σ(*I*)
Radiation source: fine-focus sealed tube	*R*_int_ = 0.027
ω scans	θ_max_ = 34.9°, θ_min_ = 1.6°
Absorption correction: multi-scan (SADABS; Sheldrick, 1996)	*h* = −12→12
*T*_min_ = 0.924, *T*_max_ = 1.000	*k* = −13→7
11665 measured reflections	*l* = −19→19
5913 independent reflections	

### Refinement

**Table:**

Refinement on *F*^2^	Primary atom site location: structure-invariant direct methods
Least-squares matrix: full	Secondary atom site location: difference Fourier map
*R*[*F*^2^ > 2σ(*F*^2^)] = 0.048	Hydrogen site location: inferred from neighbouring sites
*wR*(*F*^2^) = 0.122	H-atom parameters constrained
*S* = 1.02	*w* = 1/[σ^2^(*F*_o_^2^) + (0.0579*P*)^2^ + 0.3671*P*] where *P* = (*F*_o_^2^ + 2*F*_c_^2^)/3
5913 reflections	(Δ/σ)_max_ < 0.001
211 parameters	Δρ_max_ = 0.59 e Å^−^^3^
0 restraints	Δρ_min_ = −0.39 e Å^−^^3^

### Special details

**Table:**

Geometry. All esds (except the esd in the dihedral angle between two l.s. planes) are estimated using the full covariance matrix. The cell esds are taken into account individually in the estimation of esds in distances, angles and torsion angles; correlations between esds in cell parameters are only used when they are defined by crystal symmetry. An approximate (isotropic) treatment of cell esds is used for estimating esds involving l.s. planes.

### Fractional atomic coordinates and isotropic or equivalent isotropic displacement parameters (Å^2^)

**Table:**

	*x*	*y*	*z*	*U*_iso_*/*U*_eq_	
S1	0.52952 (4)	0.91549 (4)	0.73039 (2)	0.00972 (8)	
O1	0.70758 (13)	0.91524 (14)	0.72487 (8)	0.01414 (19)	
O2	0.51875 (14)	1.07825 (13)	0.76724 (7)	0.01422 (19)	
O3	0.45530 (14)	0.53580 (14)	0.72961 (7)	0.01357 (19)	
N1	0.42015 (15)	0.76225 (15)	0.82277 (8)	0.0107 (2)	
N2	0.36966 (17)	0.83676 (16)	0.90820 (9)	0.0138 (2)	
C1	0.38648 (17)	0.82486 (17)	0.61201 (9)	0.0097 (2)	
C2	0.44783 (17)	0.75451 (17)	0.52613 (10)	0.0103 (2)	
C3	0.32852 (18)	0.69064 (18)	0.43396 (10)	0.0116 (2)	
H3	0.367189	0.643377	0.375142	0.014*	
C4	0.15518 (18)	0.69371 (18)	0.42516 (10)	0.0126 (2)	
C5	0.09886 (18)	0.76139 (19)	0.51177 (10)	0.0133 (2)	
H5	−0.020289	0.761710	0.506665	0.016*	
C6	0.21077 (18)	0.82878 (18)	0.60579 (10)	0.0116 (2)	
C7	0.63166 (18)	0.7437 (2)	0.52408 (11)	0.0141 (2)	
H7A	0.636640	0.683730	0.456594	0.021*	
H7B	0.647194	0.675014	0.582699	0.021*	
H7C	0.730720	0.865470	0.531622	0.021*	
C8	0.0315 (2)	0.6274 (2)	0.32396 (11)	0.0190 (3)	
H8A	−0.094563	0.608096	0.337969	0.028*	
H8B	0.032099	0.513741	0.296720	0.028*	
H8C	0.076289	0.717057	0.271317	0.028*	
C9	0.1341 (2)	0.9043 (2)	0.69211 (11)	0.0190 (3)	
H9A	0.184991	1.035634	0.691039	0.028*	
H9B	0.168371	0.871735	0.761207	0.028*	
H9C	−0.000599	0.854423	0.680026	0.028*	
C10	0.40551 (17)	0.58589 (17)	0.80886 (10)	0.0099 (2)	
C11	0.32443 (17)	0.47264 (18)	0.89581 (10)	0.0106 (2)	
C12	0.30524 (18)	0.29500 (18)	0.89276 (10)	0.0132 (2)	
H12	0.343082	0.246095	0.834285	0.016*	
C13	0.23047 (19)	0.19062 (19)	0.97587 (11)	0.0153 (3)	
H13	0.216814	0.069423	0.974185	0.018*	
C14	0.17479 (19)	0.2621 (2)	1.06236 (11)	0.0160 (3)	
H14	0.123129	0.189023	1.118763	0.019*	
C15	0.19460 (19)	0.4382 (2)	1.06607 (11)	0.0154 (3)	
H15	0.157015	0.486375	1.124986	0.018*	
C16	0.27053 (18)	0.54625 (18)	0.98247 (10)	0.0118 (2)	
C17	0.2996 (2)	0.73194 (19)	0.98264 (10)	0.0145 (2)	
H17	0.264466	0.781298	1.042086	0.017*	

### Atomic displacement parameters (Å^2^)

**Table:**

	*U*^11^	*U*^22^	*U*^33^	*U*^12^	*U*^13^	*U*^23^
S1	0.00989 (13)	0.01011 (14)	0.00894 (13)	0.00401 (11)	0.00008 (10)	0.00083 (10)
O1	0.0094 (4)	0.0174 (5)	0.0147 (4)	0.0047 (4)	0.0002 (3)	0.0018 (4)
O2	0.0193 (5)	0.0103 (4)	0.0128 (4)	0.0061 (4)	0.0007 (4)	−0.0004 (3)
O3	0.0168 (5)	0.0153 (5)	0.0105 (4)	0.0083 (4)	0.0033 (3)	0.0004 (3)
N1	0.0139 (5)	0.0110 (5)	0.0083 (4)	0.0060 (4)	0.0022 (4)	0.0010 (4)
N2	0.0203 (6)	0.0139 (5)	0.0094 (4)	0.0093 (5)	0.0019 (4)	−0.0013 (4)
C1	0.0105 (5)	0.0107 (5)	0.0082 (5)	0.0046 (4)	0.0002 (4)	0.0007 (4)
C2	0.0109 (5)	0.0109 (5)	0.0106 (5)	0.0054 (4)	0.0030 (4)	0.0023 (4)
C3	0.0139 (5)	0.0121 (6)	0.0094 (5)	0.0059 (5)	0.0025 (4)	0.0006 (4)
C4	0.0121 (5)	0.0128 (6)	0.0113 (5)	0.0035 (5)	−0.0001 (4)	0.0009 (4)
C5	0.0107 (5)	0.0176 (6)	0.0130 (5)	0.0073 (5)	0.0002 (4)	0.0008 (5)
C6	0.0115 (5)	0.0143 (6)	0.0107 (5)	0.0071 (5)	0.0010 (4)	0.0005 (4)
C7	0.0118 (5)	0.0181 (6)	0.0151 (5)	0.0086 (5)	0.0035 (5)	0.0016 (5)
C8	0.0167 (6)	0.0226 (7)	0.0134 (6)	0.0050 (6)	−0.0036 (5)	−0.0036 (5)
C9	0.0186 (6)	0.0309 (8)	0.0138 (6)	0.0172 (6)	0.0002 (5)	−0.0043 (5)
C10	0.0091 (5)	0.0112 (5)	0.0094 (5)	0.0045 (4)	−0.0012 (4)	−0.0005 (4)
C11	0.0100 (5)	0.0122 (6)	0.0094 (5)	0.0044 (4)	0.0004 (4)	0.0010 (4)
C12	0.0140 (6)	0.0125 (6)	0.0132 (5)	0.0059 (5)	0.0001 (4)	−0.0003 (4)
C13	0.0143 (6)	0.0119 (6)	0.0185 (6)	0.0041 (5)	0.0009 (5)	0.0038 (5)
C14	0.0135 (6)	0.0188 (7)	0.0149 (6)	0.0052 (5)	0.0024 (5)	0.0069 (5)
C15	0.0148 (6)	0.0207 (7)	0.0116 (5)	0.0079 (5)	0.0031 (5)	0.0020 (5)
C16	0.0114 (5)	0.0137 (6)	0.0108 (5)	0.0060 (5)	0.0003 (4)	0.0006 (4)
C17	0.0195 (6)	0.0167 (6)	0.0103 (5)	0.0104 (5)	0.0025 (5)	−0.0008 (4)

### Geometric parameters (Å, º)

**Table:**

S1—O1	1.4273 (10)	C7—H7C	0.9800
S1—O2	1.4300 (10)	C8—H8A	0.9800
S1—N1	1.7422 (11)	C8—H8B	0.9800
S1—C1	1.7646 (12)	C8—H8C	0.9800
O3—C10	1.2175 (15)	C9—H9A	0.9800
N1—N2	1.3808 (15)	C9—H9B	0.9800
N1—C10	1.4003 (17)	C9—H9C	0.9800
N2—C17	1.2911 (18)	C10—C11	1.4694 (18)
C1—C6	1.4130 (18)	C11—C12	1.3943 (19)
C1—C2	1.4142 (17)	C11—C16	1.4034 (18)
C2—C3	1.3975 (18)	C12—C13	1.3847 (19)
C2—C7	1.5066 (18)	C12—H12	0.9500
C3—C4	1.3913 (19)	C13—C14	1.400 (2)
C3—H3	0.9500	C13—H13	0.9500
C4—C5	1.3883 (18)	C14—C15	1.380 (2)
C4—C8	1.5055 (19)	C14—H14	0.9500
C5—C6	1.3917 (18)	C15—C16	1.4059 (19)
C5—H5	0.9500	C15—H15	0.9500
C6—C9	1.5125 (18)	C16—C17	1.438 (2)
C7—H7A	0.9800	C17—H17	0.9500
C7—H7B	0.9800		
			
O1—S1—O2	118.39 (6)	C4—C8—H8B	109.5
O1—S1—N1	106.22 (6)	H8A—C8—H8B	109.5
O2—S1—N1	104.37 (6)	C4—C8—H8C	109.5
O1—S1—C1	112.48 (6)	H8A—C8—H8C	109.5
O2—S1—C1	110.28 (6)	H8B—C8—H8C	109.5
N1—S1—C1	103.58 (6)	C6—C9—H9A	109.5
N2—N1—C10	126.97 (11)	C6—C9—H9B	109.5
N2—N1—S1	113.93 (9)	H9A—C9—H9B	109.5
C10—N1—S1	118.89 (8)	C6—C9—H9C	109.5
C17—N2—N1	116.47 (12)	H9A—C9—H9C	109.5
C6—C1—C2	121.56 (11)	H9B—C9—H9C	109.5
C6—C1—S1	117.50 (9)	O3—C10—N1	120.86 (12)
C2—C1—S1	120.94 (10)	O3—C10—C11	124.94 (12)
C3—C2—C1	117.37 (12)	N1—C10—C11	114.19 (11)
C3—C2—C7	116.62 (11)	C12—C11—C16	120.62 (12)
C1—C2—C7	126.01 (11)	C12—C11—C10	120.00 (11)
C4—C3—C2	122.42 (12)	C16—C11—C10	119.36 (12)
C4—C3—H3	118.8	C13—C12—C11	119.31 (12)
C2—C3—H3	118.8	C13—C12—H12	120.3
C5—C4—C3	118.48 (12)	C11—C12—H12	120.3
C5—C4—C8	120.39 (12)	C12—C13—C14	120.60 (13)
C3—C4—C8	121.12 (12)	C12—C13—H13	119.7
C4—C5—C6	122.33 (12)	C14—C13—H13	119.7
C4—C5—H5	118.8	C15—C14—C13	120.31 (13)
C6—C5—H5	118.8	C15—C14—H14	119.8
C5—C6—C1	117.83 (11)	C13—C14—H14	119.8
C5—C6—C9	116.57 (12)	C14—C15—C16	119.85 (13)
C1—C6—C9	125.58 (12)	C14—C15—H15	120.1
C2—C7—H7A	109.5	C16—C15—H15	120.1
C2—C7—H7B	109.5	C11—C16—C15	119.30 (13)
H7A—C7—H7B	109.5	C11—C16—C17	117.99 (12)
C2—C7—H7C	109.5	C15—C16—C17	122.69 (12)
H7A—C7—H7C	109.5	N2—C17—C16	125.01 (12)
H7B—C7—H7C	109.5	N2—C17—H17	117.5
C4—C8—H8A	109.5	C16—C17—H17	117.5
			
O1—S1—N1—N2	120.33 (10)	C2—C1—C6—C5	−0.2 (2)
O2—S1—N1—N2	−5.52 (11)	S1—C1—C6—C5	178.53 (10)
C1—S1—N1—N2	−120.99 (10)	C2—C1—C6—C9	−178.71 (14)
O1—S1—N1—C10	−54.70 (11)	S1—C1—C6—C9	0.02 (19)
O2—S1—N1—C10	179.45 (10)	N2—N1—C10—O3	−179.88 (12)
C1—S1—N1—C10	63.98 (11)	S1—N1—C10—O3	−5.57 (17)
C10—N1—N2—C17	−0.8 (2)	N2—N1—C10—C11	0.98 (18)
S1—N1—N2—C17	−175.38 (10)	S1—N1—C10—C11	175.29 (9)
O1—S1—C1—C6	−176.04 (10)	O3—C10—C11—C12	2.1 (2)
O2—S1—C1—C6	−41.49 (12)	N1—C10—C11—C12	−178.79 (11)
N1—S1—C1—C6	69.70 (11)	O3—C10—C11—C16	−179.23 (12)
O1—S1—C1—C2	2.71 (13)	N1—C10—C11—C16	−0.13 (17)
O2—S1—C1—C2	137.26 (11)	C16—C11—C12—C13	0.6 (2)
N1—S1—C1—C2	−111.55 (11)	C10—C11—C12—C13	179.29 (12)
C6—C1—C2—C3	0.74 (19)	C11—C12—C13—C14	−0.1 (2)
S1—C1—C2—C3	−177.95 (10)	C12—C13—C14—C15	−0.3 (2)
C6—C1—C2—C7	179.99 (13)	C13—C14—C15—C16	0.2 (2)
S1—C1—C2—C7	1.30 (19)	C12—C11—C16—C15	−0.75 (19)
C1—C2—C3—C4	−0.3 (2)	C10—C11—C16—C15	−179.40 (12)
C7—C2—C3—C4	−179.65 (12)	C12—C11—C16—C17	177.93 (12)
C2—C3—C4—C5	−0.6 (2)	C10—C11—C16—C17	−0.72 (18)
C2—C3—C4—C8	178.73 (13)	C14—C15—C16—C11	0.3 (2)
C3—C4—C5—C6	1.2 (2)	C14—C15—C16—C17	−178.29 (13)
C8—C4—C5—C6	−178.16 (13)	N1—N2—C17—C16	−0.2 (2)
C4—C5—C6—C1	−0.8 (2)	C11—C16—C17—N2	0.9 (2)
C4—C5—C6—C9	177.86 (14)	C15—C16—C17—N2	179.58 (14)

### Hydrogen-bond geometry (Å, º)

**Table:**

*D*—H···*A*	*D*—H	H···*A*	*D*···*A*	*D*—H···*A*
C12—H12···O2^i^	0.95	2.49	3.3395 (18)	149

Symmetry code: (i) *x*, *y*−1, *z*.
